# Porcine Corneal Models as Translational Platforms for Innovative Therapies: Current Insights and Future Directions

**DOI:** 10.3390/jfb16120460

**Published:** 2025-12-09

**Authors:** Patrícia Nápoles, Mónica Faria, Elisa Julião Campos

**Affiliations:** 1 Department of Physics, Faculty of Sciences and Technology, University of Coimbra, R. Larga, 3004-516 Coimbra, Portugal; 2 Chemical Engineering and Renewable Resources for Sustainability (CEReS), Department of Chemical Engineering, University of Coimbra, R. Sílvio Lima, 3030-790 Coimbra, Portugal; 3 Laboratory of Physics for Materials and Emerging Technologies (LaPMET), Center of Physics and Engineering of Advanced Materials (CeFEMA), Instituto Superior Técnico, Universidade de Lisboa, Av. Rovisco Pais, 1049-001 Lisbon, Portugal; monica.faria@tecnico.ulisboa.pt; 4 Center for Neuroscience and Cell Biology, University of Coimbra, R. Larga, 3004-504 Coimbra, Portugal; 5 Centre for Innovative Biomedicine and Biotechnology, University of Coimbra, R. Larga, 3004-504 Coimbra, Portugal

**Keywords:** porcine corneal model, ex vivo model, translational platform, ophthalmic research, cell viability, drug permeability

## Abstract

The development of advanced biomaterials for corneal applications requires robust translational platforms that faithfully replicate human characteristics. Porcine corneas are increasingly recognized for ophthalmic research. Their unique combination of anatomical similarity, biomechanical comparability, and accessibility make them highly suitable for preclinical evaluation of innovative therapies, bridging the gap between preclinical discovery and clinical application. This review outlines the utility of porcine corneal models in validating advanced biomaterials, particularly in ex vivo settings, focusing on current methodologies, while addressing challenges and future directions. We aim to underscore the potential of porcine corneal models to accelerate the translation of next-generation biomaterials into clinically relevant corneal therapies.

## 1. Introduction

According to the Vision Atlas of the International Agency for the Prevention of Blindness, launched in May 2025, more than 1.1 billion people live with sight loss, despite 90% of cases being preventable or treatable. The global economic loss associated with sight loss is estimated at $411 billion each year [1]. The same report advocates for increased investment in eye health, particularly by scaling solutions in low- and middle-income countries, as this would not only improve eye health but also enhance overall well-being, learning, employment, productivity, and ultimately global socio-economic conditions.

Biomaterials have been developed to support both clinical and pharmacological approaches in ophthalmology, refining controlled drug delivery, promoting tissue regeneration, and improving surgical outcomes. Over the years, ophthalmic biomaterials have evolved, from inert structural replacements to highly adaptive, cell-instructive and biointeractive platforms. This progress has been driven by the convergence of several areas such as materials science, biomedical engineering, and ocular pathophysiology [2]. In case of corneal diseases, particular focus has been placed on developing tissues adhesives [3,4,5], artificial corneas via 3D-bioprinting and tissue engineering [6,7,8,9,10], and drug delivery systems via nanoparticles [11,12], microneedles [13], and contact lenses [14,15]. Smart contact lenses have revolutionized wearable technology with functionalities like biosensing, and drug delivery [16]. Beyond eye disease diagnosis and treatment [17,18,19], these lenses also allow mental health monitoring [20], and non-invasive diabetes controlling [21].

Following development, biomaterials require rigorous safety and efficacy evaluation to assure their suitability for real-world clinical use. The choice of experimental model is crucial to provide reliable data. Porcine models have gained significant importance in ophthalmic research. In this review, we highlight the growing relevance of porcine corneas as robust preclinical platforms for eye research. Their unique combination of anatomical and physiological similarities to human tissue, biomechanical comparability, and accessibility makes them ideal for assessing innovative biomaterials and therapeutic strategies. We provide a comprehensive synthesis of current methodologies employing porcine corneal models, particularly in ex vivo settings, and evaluate their strengths, current limitations, and future opportunities.

## 2. Anatomy of the Human Eye

The human eye contains three chambers: the anterior chamber, located between the cornea and the iris and filled with aqueous humor; the posterior chamber, located between the iris and lens, and also containing aqueous humor [22]; and the vitreous chamber, which lies behind the lens adjacent to the retina and is filled with a gel-like vitreous humor.

The eye is also structurally organized in (i) the outer fibrous layer, consisting of the cornea and sclera, which provides protection and maintains the shape of the globe; (ii) the middle vascular layer, which includes the iris (regulating light entry through the pupil), the ciliary body (producing aqueous humor), and the choroid (supplying nutrients to the outer retina); and (iii) the inner neural layer—the retina—, which contains neural cells that convert light into electrical signals [22,23].

### 2.1. Cornea

**Figure 1:**
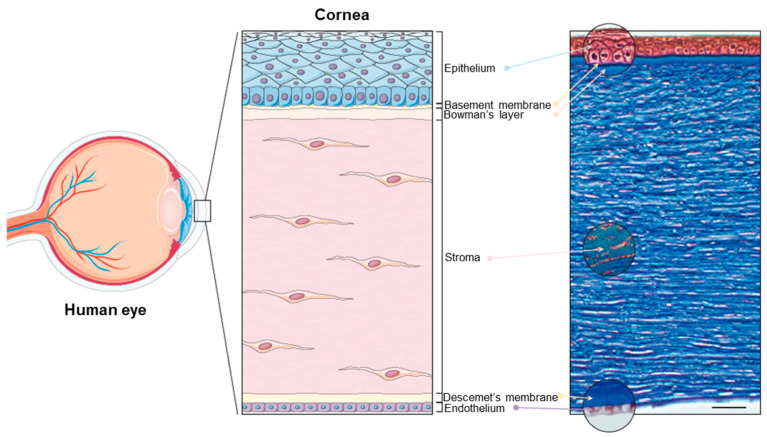
The human cornea. (**Left panel**): Schematic diagram of the human eye, highlighting the cornea. (**Right panel**): Masson trichrome staining showing the layers of the cornea: the epithelium (40–50 μm), the basement membrane (0.1–0.6 μm), the Bowman’s layer (8–15 μm), the stroma (470–500 μm), the Descemet’s membrane (10–12 μm), and the endothelium (4–6 μm). Scale bar: 50 μm. Reprinted from Ref. [25].

The cornea is the principal refracting component of the eye (43.25 diopters (D) out of a total of 58.60 D) [22,24]. Its transparency and avascularity provide optimal light transmittance. From anterior to posterior, the layers that compose the cornea are the epithelium, the (epithelial) basement membrane, the Bowman’s layer, the stroma, the Descemet’s membrane, and the endothelium (Figure 1).

**Figure 2:**
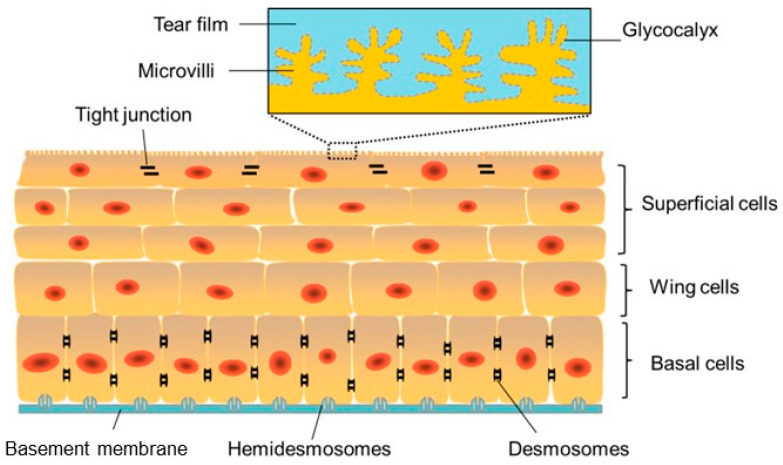
Schematic image of the corneal epithelium showing adhesion between cells and to the under lying basement membrane (blue) and the Bowman’s layer via hemidesmosomes. Reprinted from Ref. [26].

The corneal epithelium is a lipophilic, nonkeratinized, stratified epithelium consisting of 4–6 cell layers [22] (Figure 2).

The superficial layer, which is 1 to 2 cells thick, consists of flattened squamous cells with tight junctions between these cells. Tight junctions form a crucial barrier that prevents intercellular passage of substances and controls fluid uptake from the tear film, creating a selective semi-permeable membrane. Desmosomes provide additional adhesion, reinforcing this barrier. The middle layer consists of 2 to 3 layers of wing cells, interconnected by desmosomes and gap junctions.

The Bowman’s layer consists of a dense, acellular sheet of randomly arranged collagen fibrils, serving as a transition zone between the epithelium and the stroma. Although its exact function remains unclear, it is believed to contribute to the biomechanical rigidity and shape of the cornea.

The corneal stroma accounts for approximately 90% of the total corneal thickness [22]. It is primarily composed of collagen fibrils (mainly type I with some type V), keratocytes, and extracellular ground substances. The collagen fibrils are arranged in regularly spaced, parallel lamellae extending from limbus to limbus. This precise organization is essential to maintain corneal transparency [24].

The Descemet’s membrane is continuously secreted by endothelial cells and gradually thickens with age. This layer is particularly rich in type IV collagen.

The corneal endothelium is a single layer of flattened, typically hexagonal cells located adjacent to the anterior chamber. This hexagonal arrangement, known as the endothelial mosaic, maximizes surface coverage and structural efficiency. Endothelial cells have limited proliferative capacity in humans, so their number gradually declines with age (from 3000–4000 cells/mm^2^ in children to 1000–2000 cells/mm^2^ by the age of 80) due to cell loss and disintegration [22,24].

Interestingly, the corneal layers exhibit different responses to injury. Damage to the epithelial layer heals through the migration of epithelial cells generated at the limbus. In case the injury affects the Bowman’s layer, it is replaced by scar tissue [27]. Injury to the stroma activates keratocytes, which transition into repair-type stromal fibroblasts and contractile myofibroblasts; these cells overproduce extracellular matrix in a disorganized arrangement. The resulting scar formation may lead to corneal opacities, blocking light penetration, and causing visual impairment [28]. Injury to the endothelium disrupts its normal function, leading to corneal edema due to the loss of the sodium-potassium pump function of the cells, with subsequent visual impairment [29].

### 2.2. Ocular Barriers

The presence of ocular barriers is the key challenge for therapeutics in terms of reaching the target site and remaining there for a sufficient duration. Particularly, the corneal epithelium and the tear film act as selective barriers, regulating molecular transport and protecting against external pathogenic agents. These barriers are particularly important in the context of topical ocular drug delivery, as they influence the ability of therapeutic agents to reach specific target tissues, such as the cornea.

The tear film constitutes a dynamic barrier to drug administration mainly because of continuous tear turnover and nasolacrimal drainage. In fact, following topical drug administration, tear turnover increases significantly, promoting rapid clearance of the drug via nasolacrimal drainage often within one to two minutes. Due to the limited ocular surface area most of the solution (typically ~30 µL) is quickly lost. It is estimated that around 60% of the drug is eliminated within the first 2 min. After 8 min, the drug concentration on the ocular surface may drop to 0.1%, and, after 15 to 25 min, nearly all active components are cleared [30]. This dynamic clearance substantially reduces drug bioavailability and limits therapeutic efficacy in ocular treatments.

**Figure 3:**
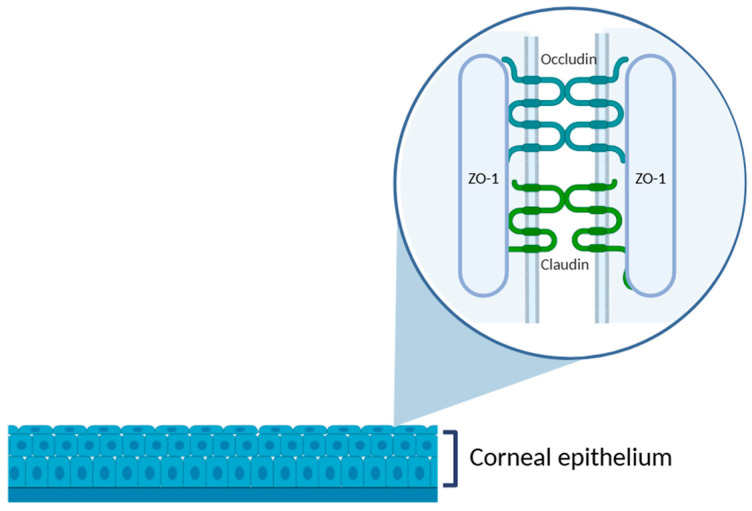
Schematic representation of the corneal epithelium, highlighting the structure of tight junctions in the superficial epithelial layer. The zoomed-in area illustrates the intercellular junctional complex composed of transmembrane proteins, such as occludin and claudins, and the cytoplasmic scaffold protein ZO-1, which anchors the complex to the actin cytoskeleton. These proteins are essential to maintain the epithelial barrier function and regulate paracellular permeability. Illustration created with BioRender (version 04).

The cornea is an anatomical barrier to the penetration of topically applied drugs into intraocular structures. As the outermost corneal layer, the epithelium represents the most substantial barrier to drug absorption. The tightly packed epithelial cells are interconnected by various intercellular junctions: tight junctions, adherens junctions, desmosomes, and gap junctions. Among these, tight junctions, which are located predominantly in the superficial epithelial layer, are particularly critical to maintaining the structural and functional integrity of the corneal barrier. They are composed of transmembrane proteins, such as occluding, and members of the claudin family, which are anchored intracellularly by scaffold proteins like *zonula occludens*-1 (ZO-1) [31] (Figure 3).

This complex and dynamic architecture plays a pivotal role in preserving epithelial homeostasis and protecting the underlying ocular tissues. As a result, the presence of tight junctions constitutes a major challenge to effective topical delivery, often resulting in limited bioavailability.

## 3. Experimental Models in Corneal Research

The study of the cornea and its pathologies depends on reliable experimental models that enable the investigation of physiological mechanisms, the testing of novel therapeutic formulations, and the assessment of the safety and efficacy of biomaterials or ophthalmic drugs before clinical application. Due to the complex and stratified nature of the cornea, the choice of an appropriate experimental model is essential to ensure relevant and translational results. In vision research, in vitro, ex vivo, and in vivo approaches are all employed, with animal models playing a central role in each one of these approaches [32].

### 3.1. In Vitro, Ex Vivo, In Vivo Corneal Models

Over the past decades, various in vitro, ex vivo, and in vivo models have been developed to reproduce, in a controlled manner, the physiological conditions of the human cornea.

**In vitro corneal models** use cultured cells or reconstructed tissues that provide a controlled environment for studying cellular behavior, molecular mechanisms, and tissue responses. They enable precise manipulation of experimental conditions and offer high reproducible, making them valuable for investigating corneal physiology, drug permeability, toxicity, and wound healing processes [33,34,35]. Recently, advances in 3D cultures and corneal organoids have significantly improved their physiological relevance [36].

Corneal cell monolayer models represent one of the simplest and most widely used in vitro approaches. In these models, a single layer of corneal epithelial, stromal, or endothelial cells is cultured on a suitable substrate, allowing researchers to analyze cellular behavior under controlled conditions [37,38]. A study by Travers et al. [39] investigated the role of molecular coatings in human corneal endothelial cell monolayers. Zhang et al. [33] utilized monolayer cultures of corneal epithelial cells to examine how the ZEB1 gene influences cell migration, showing that ZEB1 plays a key role in promoting epithelial migration, a process essential for corneal wound healing.

3D corneal models and organoids have been developed to more accurately reproduce the structural and functional complexity of the human cornea. Their underlying principle is to recreate the native three-dimensional, particularly cell–cell and cell–matrix interactions, by arranging one or more corneal cell types within scaffolds, hydrogels, or bioprinting-derived matrices that mimic the biochemical and mechanical properties of the extracellular matrix [36]. Compared with monolayer cultures, which primarily capture two-dimensional cellular behavior, these advanced systems better preserve tissue stratification, spatial organization, and physiological signaling gradients, thereby enabling more realistic assessments of corneal function. Organotypic epithelial cell models have been developed to study the effects of topical drugs on the corneal epithelium. In these models, primary epithelial cells are cultured on membrane inserts and exposed to an air–liquid interface, thereby approximating in vivo physiological conditions. The reconstructed human corneal epithelial model (EpiOcular™, MatTek Life Sciences, Ashland, MA, USA), for example, consists of human corneal epithelial cells grown into a stratified squamous epithelium using 3D culture condition [35]. Da Silva et al. [34] applied this model as a screening tool for evaluating ocular toxicity potential of isolated chemicals and botanical mixtures, demonstrating its utility in predictive toxicology. Compared with monolayer systems, 3D models and organoid offer more physiologically relevant cell–cell and cell–matrix interactions, allowing more accurate simulation of wound healing, drug penetration, and barrier properties, and disease-specific phenotypes [36]. However, this added complexity introduces trade-offs: these models are more technically demanding and are generally less reproducible than simple monolayer cultures. Standardization across laboratories remains a challenge that currently limits widespread adoption [9].

**Corneal 3D bioprinting** offers a promising solution by enabling the precise, layer-by-layer fabrication of corneal tissues, closely mimicking the essential characteristics needed for corneal substitution, vision restoration, and long-term graft success. A significant advancement in bioprinting is the development of multi-material bioprinters, which facilitate the creation of multicellular structures with precise spatial arrangement [9,40]. However, the transition from laboratory design to large-scale production necessitates the classification and regulation of these product types, accompanied by the establishment of specific legislation designed to ensure their safety [41].

Organ chip technology, particularly **cornea chip models** [42], has emerged as a promising alternative for studying corneal physiology, pathology, and drug screening. Current cornea chips enable researchers to investigate drug transport, barrier integrity, and disease mechanisms with improved physiological relevance, such as bacterial keratitis [43] and dry eye [44]. Nevertheless, many challenges exist, namely recapitulating the extracellular matrix organization, with its unique composition and arrangement of collagen fibrils, proteoglycans, and glycoproteins, crucial role to maintain corneal transparency, biomechanical properties, and cellular behavior. Also, the complex interactions between corneal epithelial, stromal, and endothelial cells, as well as their surrounding microenvironment, essential for maintaining corneal homeostasis and wound healing, need further improvements [42].

**Ex vivo corneal models** serve as an essential bridge between simplified in vitro systems and complex in vivo studies. Their core principle lies in the use of intact corneal tissues obtained from human donors, animals, or abattoir sources that preserve the native architecture, cellular stratification, and biochemical composition of the cornea. By preserving the structural and functional features of corneal layers, ex vivo models offer a level of biological realism not achievable in monolayer or reconstructed in vitro systems. This allows for the assessment of drug permeability, wound healing, and tissue responses under conditions that closely mimic the in vivo environment. Several recent studies have explored a wide range of ex vivo platforms for ophthalmic research [45,46].

Barbalho et al. [47] developed a dynamic ex vivo porcine eye model incorporating simulated tear flow to evaluate ophthalmic drug penetration, providing more realistic pharmacokinetic assessments. Pescina et al. [48] established an ex vivo porcine corneal model integrating histological analysis with permeability testing to investigate how physicochemical properties (particularly compounds hydrophilicity) influence transcorneal transport. Shi et al. [49] utilized an ex vivo porcine cornea model to study *Acanthamoeba* infections, demonstrating its suitability for infectious disease research. Similarly, Okurowska et al. [50] used a porcine ex vivo cornea model to investigate antimicrobial therapies for *Pseudomonas aeruginosa*-induced keratitis, providing a reproducible setting for testing treatment efficacy under physiologically relevant conditions. Tsai et al. [51] established a human corneal endothelium wound model using donor corneas maintained in an anterior chamber perfusion system, allowing continuous monitoring of corneal thickness, and evaluation of endothelial healing responses to cell-based or pharmacological therapies. Castro et al. [52] investigated stromal and epithelial regeneration by culturing porcine corneas following anterior keratectomy, providing valuable insight into fibrosis pathways and myofibroblast activation mechanism. Netto et al. [53] reproduced the inflammatory and structural features of dry eye disease by incubating porcine corneas at low humidity, highlighting the potential of ex vivo platforms for studying ocular surface disorders. In another study, Rouhbakhshzaeri et al. [54] designed a new ex vivo porcine model to mimic endothelial injury caused by phacoemulsification and tested the therapeutic potential of mesenchymal stromal cell secretome, as a regenerative and protective effects on the corneal endothelium. Rodrigues da Penha et al. [55] developed a bovine ex vivo model for chemical toxicity assessment, successfully distinguishing ocular irritant compounds, and supporting its applicability in cosmetic safety testing. Sarfraz et al. [56] employed ex vivo porcine eyes to evaluate a twin nanoparticulate drug delivery system designed to enhance dexamethasone permeation and ocular retention, confirming the relevance of ex vivo approaches for preclinical screening of advanced formulations.

Ex vivo corneal models offer notable advantages that make them valuable tools for ophthalmic research. They preserve native corneal architecture, barrier properties and tissue-specific responses, enabling realistic assessment of drug permeability, wound healing, infection dynamics, and toxicity under controlled and physiological conditions. Furthermore, the use of porcine or bovine corneas from abattoir sources provides a cost-effective and ethically preferable alternative to in vivo animal testing [46]. However, despite their translational relevance, these systems also present significant limitations. Tissue viability decreases over time in the absence of systemic circulation, limiting experiments to a few days [57]. Variability among donor tissues and difficulties in reproducing immune or vascular responses further constrain their predictive accuracy for chronic or systemic effects [50,58]. Consequently, while ex vivo corneal models effectively bridge the gap between in vitro and in vivo experimentation, they are best suited for short-term studies of drug permeability, toxicity, or early-stage therapeutic screening.

**In vivo corneal models** use live animals to study corneal physiology, disease mechanisms, therapeutic efficacy, and safety within the complexity of an intact biological system. Their fundamental principle is the ability to capture integrated physiological responses that ex vivo or in vitro systems cannot fully recapitulate, such as immune response, vascularization, metabolism, and systemic pharmacokinetics. Importantly, the primary purpose of an animal model is not to replicate human conditions in every detail, but rather to reproduce the specific aspects most relevant to for understanding a disease or testing a therapeutic intervention.

A wide range of vertebrate species is used depending on the research question, including rodents, rabbits, pigs, primates, felines, and canines, as well as invertebrates that include flies and nematodes. The selection of an appropriate model requires careful consideration, as differences in ocular anatomy, physiology, and drug response can significantly affect experimental outcomes. For instance, variations in corneal thickness, eye axial length, and ocular surface properties between species can influence drug absorption, distribution, and overall treatment efficacy (Table 1). These interspecies differences have direct implications on experimental design and translation. For instance, rodent corneas, though useful for genetic studies, differ markedly in size and barrier properties from human corneas, while rabbit and porcine eyes more closely approximate human anterior segment anatomy. Understanding these comparative features is therefore essential for accurately interpreting data and for determining how well findings can be extrapolated to human applications [32].

In vivo corneal models have been extensively used to study therapeutic delivery, drug bioavailability, and molecular mechanisms of wound healing. Peterson et al. [66] employed a rabbit corneal suture model to evaluate the sustained delivery of bevacizumab using biodegradable densomere microparticles, showing how in vivo models can be applied to assess long-term drug release and pharmacodynamic effects. Chauchat et al. [67] used pigmented rabbits to compare the ocular bioavailability of several latanoprost formulations, showing the value of in vivo models to study the influence of formulation excipients on the pharmacokinetics. Similarly, Chen et al. [68] used a C57BL/6 mouse corneal injury model to investigate epithelial wound healing and the role of interleukin—36 receptor signaling, highlighting the ability of in vivo models to study the molecular pathways involved in tissue repair. Akpek et al. [69] used a rabbit model to test the integration and functional performance of a second-generation synthetic cornea, illustrating how in vivo systems can provide critical insights into biocompatibility under physiological conditions. Yamashita et al. [70] similarly employed rabbit models to study corneal endothelial dysfunction, enabling detailed study of disease mechanisms and the preclinical evaluation of potential therapeutic strategies. Additionally, Sun et al. [71] used in vivo models for studying specific corneal pathologies, such as Fuchs’ endothelial corneal dystrophy, allowing to investigate disease progression and treatment effects in a whole-organism context. Koseoglu et al. [72] used advanced in vivo imaging techniques, combined with deep learning to diagnose neuropathic corneal pain, highlighting the role of in vivo models in developing and validating novel diagnostic tools. Collectively, these examples emphasize the versatility of in vivo corneal models in preclinical ophthalmic research, from drug delivery and pharmacokinetics to mechanistic studies of corneal physiology and pathology in a biologically relevant environment. Their ability to capture integrated physiological responses makes them indispensable for translating laboratory findings into clinically relevant insights.

Despite their strong relevance in translational research, in vivo corneal models present several limitations. Anatomical and physiological differences between animal models and the human eye, such as corneal thickness, endothelial density, stromal organization, and immune responses, can complicate the extrapolation of findings and reduce predictive accuracy for human applications [32]. Ethical concerns and increasing regulatory restrictions also restrict the use of live animals, especially in large-scale or long-term studies [73]. Biological variability among individual animals can also increase data heterogeneity, often necessitating larger sample sizes to achieve statistical power. Furthermore, in vivo experiments are costly, time-consuming, and require specialized facilities and expertise, which may constrain experimental design and reproducibility [74]. Consequently, while in vivo models remain indispensable for capturing integrated organism-level responses, their limitations underscore the need to wisely select species and precede in vivo findings by ex vivo and in vitro approaches to strengthen translational validity.

### 3.2. Porcine Corneal Models

The porcine eye is widely used as a model in vision science due to its anatomical and physiological similarity to the human eye [63]. The overall globe size, general ocular morphology, and retinal vasculature pattern closely resemble those of the human eye. Also, the absence of a *tapetum lucidum* (a reflective layer of the retina where retinal pigment epithelium (RPE) cells are not pigmented), makes it more comparable to the human RPE than that of other farm animals, such as cows or sheep [65]. More detailed comparative parameters between porcine and human eyes are summarized in Table 2.

Porcine eyes can be used both as in vivo and ex vivo models; however, they are mostly used in ex vivo corneal research due to practical and ethical considerations. Conducting ophthalmic experiments in live pigs presents several challenges, including rapid animal growth that complicates long-term studies, limited orbital space that complicates surgical procedures, and high costs associated with housing and handling [32]. These factors may reduce the feasibility of porcine in vivo models in routine or large-scale research. In contrast, ex vivo porcine eyes are readily available as by-products of the food industry. Since ex vivo porcine eyes are collected *post-mortem* from slaughterhouses, their use does not involve the deliberate sacrifice of animals for research purposes. This accessibility not only reduces ethical concerns but also supports high experimental throughput and reproducibility. As a result, ex vivo porcine models offer a cost-effective, accessible, and ethically responsible alternative that still retains strong anatomical and physiological relevance to the human eye [77].

Porcine eye models have been applied across a wide range of ophthalmic research areas, including studies of the neurosensory retina, cataract surgery research, corneal transplantation, aberrometry, and transscleral drug delivery [63,77,78]. Ex vivo porcine corneal models are particularly valuable for assessing the safety and potential toxicity of chemical and pharmaceutical compounds before proceeding to in vivo or clinical testing [79]. Their anatomical similarity to the human eye and wide availability, also makes them an excellent platform for surgical training. Indeed, porcine eyes are routinely used by ophthalmology residents to practice and refine surgical techniques, and many corneal procedures and innovations have been first developed and optimized using ex vivo porcine ocular tissues [80,81].

Despite their many advantages, ex vivo porcine corneal models also present limitations that must be considered when interpreting experimental results. One of the main drawbacks is the absence of physiological factors such as blood flow, tear film dynamics, and immune responses, all of which influence drug absorption, metabolism, and tissue repair in vivo. *Post-mortem* tissue degradation and variability in the time between eye collection and experimentation can further affect corneal integrity and cellular viability, introducing additional sources of variability. The lack of standardized collection and preservation protocols across studies further contributes to experimental variability. Nevertheless, porcine models remain highly valuable translational tools, effectively bridging the gap between in vitro experimentation and human ophthalmic applications.

## 4. Current Methodologies

### 4.1. Cell Viability

Assessing cell viability is a crucial step in validating and supporting ocular therapy research. The evaluation of cell viability provides valuable insights into tissue health following experimental manipulation, drug exposure, surgical procedures, or storage conditions [54,82]. It also serves as a key indicator of safety and biocompatibility before advancing to in vivo studies or clinical trials. Therefore, assessing the cytotoxic potential of formulations, biomaterials, or surgical interventions in ex vivo porcine corneas yields essential preclinical data that contributes to the refinement of ocular therapies under physiologically relevant yet ethically sustainable conditions. In ex vivo porcine corneas, viability is commonly assessed in specific layers depending on the research purpose. Epithelial, endothelial and keratocyte viability is typically evaluated to examine the effects of formulations under research, toxic or mechanical injuries.

**Figure 4:**
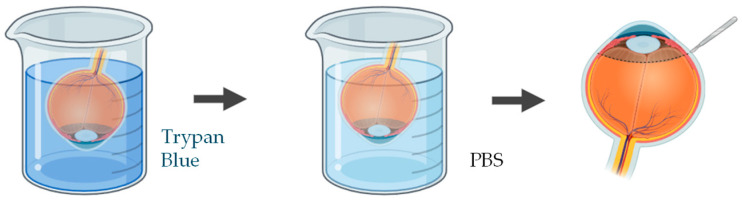
Schematic representation of the procedure used to assess epithelial cell viability in ex vivo porcine corneal models, using trypan blue. Illustration created with BioRender (version 04).

A wide range of studies have been applied to quantify and visualize viable and non-viable cells in porcine ex vivo models. To evaluate corneal epithelial cell viability, the most used approach involves vital dye staining with trypan blue. Following the experimental procedure, the whole cornea is immersed in a 0.4% trypan blue solution for approximately 2 min, with the epithelial surface facing downward. The tissue is then gently rinsed in phosphate-buffered saline (PBS) to remove excess dye. Subsequently, the cornea is carefully dissected to isolate it, ensuring minimal mechanical damage (Figure 4). Microscopic examination is performed to quantify the number of trypan blue–stained cells, which correspond to non-viable epithelial cells [83,84]. This method provides a rapid and reliable estimation of epithelial integrity after topical drug exposure or mechanical stress.

**Figure 5:**
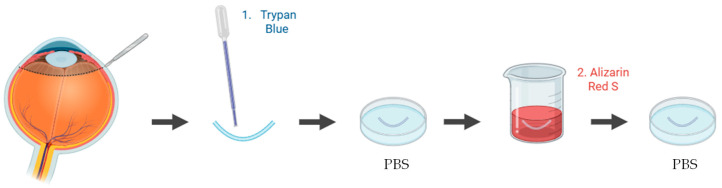
Schematic representation of the procedure used to assess endothelial cell viability in ex vivo porcine corneal models, using trypan blue and alizarin red S staining. Illustration created with BioRender (version 04).

To assess endothelial cell viability in ex vivo porcine corneal models, several vital staining methods have been applied, most commonly using trypan blue and/or alizarin red S. In one study, the endothelium was exposed to 0.2% trypan blue, and the proportion of blue-stained non-viable cells was quantified relative to the total endothelial surface [85]. Another study employed alizarin red S to evaluate endothelial morphology and detect alterations in cell membranes [86]. In a different approach, both trypan blue and alizarin red S were combined to enhance the visualization of cell borders and improve the assessment of endothelial morphology and viability [54] (Figure 5). Alternatively, fluorescence-based Live/Dead assays using calcein-acetoxymethyl ester (AM) and ethidium homodimer-I was used to simultaneously identify viable and non-viable cells [87]. Collectively, these techniques provide reproducible information on endothelial integrity, which is a critical parameter to validate the safety of ocular therapies.

To evaluate keratocyte viability in an ex vivo porcine corneal model, keratocytes were labeled with the fluorescent probe 5-chloromethylfluorescein diacetate (CMFDA) and visualized by confocal laser scanning microscopy, allowing for high-resolution imaging of viable stromal cells and assessment of tissue integrity [88]. This approach provided detailed morphological information.

### 4.2. Drug Permeability

The evaluation of corneal drug permeability is a crucial step in the preclinical development of ophthalmic formulations, as it directly influences the therapeutic efficacy of ocular treatments. Porcine eyes are one of the most suitable models for drug diffusion and permeability studies because of their close resemblance to human eyes in terms of globe size, vascular anatomy, histological features, physiological properties, and stromal collagen bundle organization. The porcine cornea presents a comparable overall thickness and an identical endothelial thickness to that of humans. However, notable structural differences exist: the porcine corneal epithelium and sclera are roughly twice as thick as those in humans, and the corneal stroma is about 30% thicker [45]. These variations must be carefully considered when interpreting results from permeation experiments.

**Figure 6:**
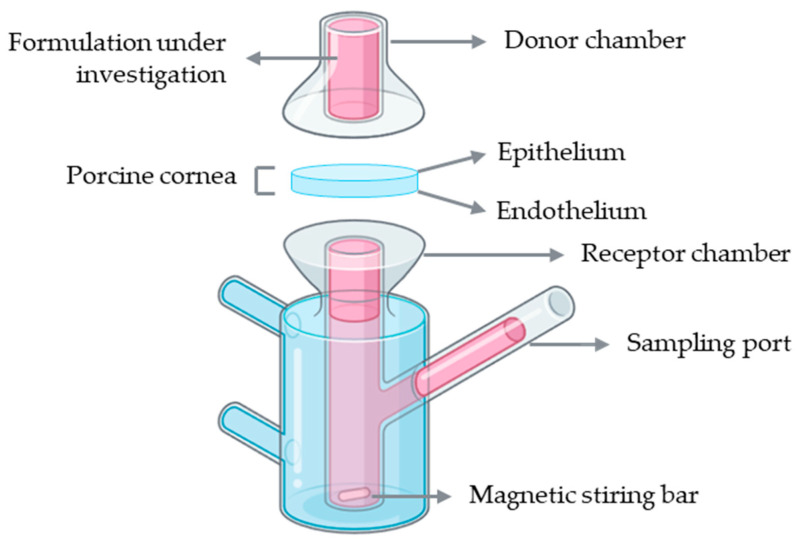
Schematic representation of a Franz diffusion cell setup using an ex vivo porcine cornea for drug permeability assessment. Illustration created with BioRender (version 04).

Franz diffusion cells represent the most widely used setup for ex vivo corneal permeability studies. A conventional Franz diffusion system is composed of two compartments: a donor and a receptor chamber separated by a membrane, which in this case corresponds to the porcine cornea with the epithelial surface facing the donor chamber to simulate topical drug application (Figure 6). The donor chamber receives the formulation under investigation, while the receptor chamber, connected to a sampling port, allows periodic collection of samples for quantitative analysis. The receptor compartment is typically filled with a physiologically relevant buffer solution, such as PBS or simulated tear fluid, maintained at 37 °C and continuously stirred with a magnetic stirrer to ensure homogeneity. At predefined intervals, aliquots are collected from the receptor chamber, and the amount of permeated drug is quantified using analytical methods such as high-performance liquid chromatography (HPLC) or ultraviolet–visible (UV–Vis) spectrophotometry [45].

Several studies have employed Franz diffusion cells with ex vivo porcine corneas to investigate drug permeability across ocular tissues. For instance, this model has been used to assess the permeation of erythropoietin, desmoteplase, and carprofen, as well as to evaluate novel ophthalmic formulations such as ciprofloxacin polymeric films and riboflavin-5′-phosphate enhanced with vitamin E D-α-tocopheryl polyethylene glycol succinate (TPGS) [89,90,91,92,93]. These examples highlight the versatility of the Franz diffusion cell setup for studying transcorneal drug diffusion and comparing the permeability profiles of different compounds and formulations. Despite their widespread use in corneal permeability studies, Franz diffusion cells present several limitations. The system only supports vertical diffusion and fails to reproduce the natural curvature and physiological environment of the cornea. Also, unintentional corneal damage can be generated in this model. Furthermore, prolonged exposure of the endothelium to the receptor medium may also cause stromal swelling due to excessive hydration, further affecting the accuracy of permeability measurements [94].

Whole-eye models use intact porcine eyes to study corneal drug permeability under more physiologically relevant conditions than isolated corneal setups such as Franz diffusion cells. By preserving the natural curvature and anatomical relationships of the eye, these models allow for drug penetration to be assessed in a context that closely mimics in vivo conditions. Drug formulations are applied to the corneal surface, and samples are collected from the aqueous humor or other compartments to determine penetration. Although the basic setup is the same, experimental protocols can be adapted depending on the drug, the detection method or the specific goals of the study. For instance, Barbalho et al. [47] developed a dynamic model with simulated lacrimal flow to evaluate the performance of pharmaceutical drug products. Similar, Bhujbal et al. [95] assessed the ocular drug penetration using ex vivo porcine whole-eye model with simulated tear flow. Sun et al. [96] investigated the permeation dynamics of organosilica nanoparticles across porcine corneal barriers for glaucoma drug delivery. Sarfraz et al. [56] also carried out an ex vivo whole eye permeation study. While these models provide more realistic diffusion dynamics, they are limited by variability between donor eyes, tissue viability over time, and the absence of systemic circulation.

**Figure 7:**
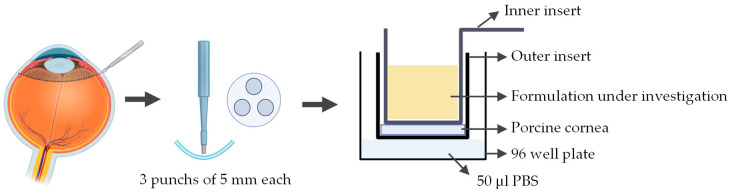
Schematic representation of the procedure used to assess corneal permeability to a formulation under investigation using the ex vivo cell insert model. Illustration created with BioRender (version 04).

A novel ex vivo corneal permeability model has been developed using CellCrown™ inserts in a 96 well plate format, enabling high-throughput testing while reducing the number of porcine eyes required [97]. 5 mm corneal punches are placed into the base of the outer insert with the epithelium facing upwards. The inner insert is positioned on top of the corneal disk to create a water-tight seal without deforming the tissue. The assembled inserts are placed into the well of a 96 well plate containing PBS, with parafilm or a similar cover used to prevent evaporation. To assess the permeability the study substance was pipetted into the inner insert (Figure 7). Despite allowing for high throughput testing, this ex vivo model has several limitations. The use of small corneal punches fails to reproduce the natural curvature of the cornea. Variability between porcine eyes and challenges in maintaining tissue viability can also impact reproducibility.

Even though corneal permeability has been extensively investigated in numerous studies, the underlying causes of increased permeability are often not explored in detail. Understanding why permeability changes occur is essential for elucidating the molecular mechanisms that take place in the ocular surface following the application of ophthalmic therapies. Ophthalmic therapies, such as drug formulations or contact lenses may alter corneal barrier properties, but without studying the specific pathways involved, it remains unclear whether these effects are transient, reversible, or associated with epithelial damage. Therefore, identifying the cellular and molecular basis behind changes in permeability is a critical step toward developing safer ocular treatments.

Tight junctions play a key role in maintaining the structural and functional integrity of the corneal barriers. Tight junctions are composed of transmembrane proteins, such as occludin and members of the claudin family, which are anchored intracellularly by scaffold proteins, such as ZO-1 [31]. Theoretically, an increase in corneal permeability is directly related to a loss of tight junction integrity and, consequently, disruption of the epithelial layer [98]. Assessing tight junctions’ integrity thus provides a mechanistic link between observed permeability changes and cellular barrier function. Some studies have investigated tight junction alterations using immunofluorescence labeling in ex vivo rabbit and mouse corneal models [99,100,101,102]. However, no equivalent data currently exists for porcine models. Considering the structural and physiological similarities between porcine and human corneas, performing such analyses in the porcine model would provide valuable insights into drug-induced barrier modulation and enhance translational relevance in preclinical testing.

## 5. Concluding Remarks and Future Perspectives

Porcine ex vivo corneal models represent a powerful translational platform in preclinical ophthalmic research. Their anatomical and physiological similarity to human cornea, combined with their consistent availability from abattoir sources, provides an ethically advantageous, cost-effective, and reproducible alternative to in vivo models. These features position porcine tissues as an ideal system for mechanistic studies, therapeutic screening, and methodological development.

Despite these advantages, research using porcine ex vivo corneas remain relatively limited. A major challenge is the absence of standardized protocols to assess key corneal features, such as epithelial barrier integrity. Critical proteins like ZO-1 and occludin, which play essential roles in barrier function, have not been evaluated in porcine ex vivo corneas, representing a notable knowledge gap. We identified this critical gap and successfully developed a protocol to address it. We are currently preparing a manuscript presenting this protocol, along with additional methodologies using porcine ex vivo corneas, to facilitate cross-laboratory harmonization and elevate the scientific rigor of future porcine-based studies.

Looking ahead, porcine ex vivo corneas represent an exceptionally valuable translational model. Their structural and physiological similarity to human eyes, versatility, and wide availability, allow the use in preclinical ophthalmic research. This model allows for the integration of diverse techniques, including permeability assays, cell viability assessments of epithelium, endothelium, and keratocytes, as well as evaluations of tight junction integrity. Standardizing these methodologies can greatly enhance reproducibility across laboratories and facilitate broader adoption. By capitalizing on these inherent advantages, researchers can not only bridge existing knowledge gaps but also accelerate the development of safe and effective ocular therapies, while minimizing reliance on live animals.

Artificial intelligence (AI) is expected to play a critical role in biomaterials field, not only in the development but also in verifying their efficacy across a wide range of applications. When integrated with animal models, such as porcine models, AI can increase the efficiency of preclinical research by finding patterns in complex biological data, helping researchers understand that data more easily, and generating new hypotheses faster and on a larger scale. At the same time, AI offers a promising opportunity to reduce animal use in biomaterials evaluation, paving the way for animal-free research.

Summing up, porcine ex vivo corneas represent a robust, practical, and translationally relevant platform for vision sciences research. With coordinated efforts toward protocol standardization and validation, these models can become a cornerstone in the translational pipeline, bridging foundational research and clinical innovation with greater precision, reproducibility, and ethical responsibility.

## Figures and Tables

**Table 1:** Comparison of ocular parameters between the human and relevant laboratory animal species. The values presented are average measurements reported in the literature. It is worth noting that several factors, such as age, physiological condition, and the measurement method, can significantly influen8ce these parameters.

	Mouse	Rat	Rabbit	Porcine	Human
Average eye dimension in volume (cm^3^)	0.025	0.1	2.6	6.5	7.2
Average eye dimension (axial length in mm)	3.4	6.0	17.1	23.9	24
Corneal horizontal diameter (mm)	3.15	5.1	13.4	14.3	11.81
Corneal thickness (µm)	0.089–0.123	0.16–2	0.36	543–797	530–710
Cornea shape	Flatter	Flatter	Dome	Dome	Dome
Bowman’s layer thickness (µm)	Yes (0.7–0.8)	Yes (1)	Yes (3)	Yes (0.94)	Yes (10)
Time between eye blinks	5 min	5 min	6 min	20–30 s	5 s
References	[32,59,60,61,62]			[32,59,60,61,63,64]	[23,32,59,60,61,62,65]

**Table 2:** Comparison of ocular parameters between the porcine and human eye. The values presented are average measurements reported in the literature. It is important to note that several factors, such as age, physiological condition, and the measurement method, can significantly influence these parameters.

	Porcine	Human
Corneal curvature	7.85–8.28 mm [75]	6.5–7.8 mm [24]
Corneal epithelium thickness	80 μm [63]	50 μm [24]
Corneal epithelium cell layers	6–8 layers [76]	4–6 layers [22]
Corneal endothelium cell density	3250 cell/mm^2^ [75]	2496.9–4049.5 cell/mm^2^ [75]
Retinal thickness	300 μm [65]	310 μm [65]

## Data Availability

No new data were created or analyzed in this study. Data sharing is not applicable to this article.

## Abbreviations

The following abbreviations are used in this manuscript:

AM	Acetoxymethyl ester
CMFDA	5-chloromethylfluorescein diacetate
D	Diopter
HPLC	High-performance liquid chromatography
PBS	Phosphate-buffered saline
RPE	Retinal pigment epithelium
TPGS	D-α-tocopheryl polyethylene glycol succinate
UV–Vis	Ultraviolet–visible
ZO-1	*Zonula occludens*-1
